# MicroRNA-221/222 Inhibits the Radiation-Induced Invasiveness and Promotes the Radiosensitivity of Malignant Meningioma Cells

**DOI:** 10.3389/fonc.2020.01441

**Published:** 2020-08-25

**Authors:** Qing Zhang, Lai-Rong Song, Xu-Lei Huo, Liang Wang, Guo-Bin Zhang, Shu-Yu Hao, Hai-Wei Jia, Chui-Lin Kong, Wang Jia, Zhen Wu, Bai-Nan Xu, Gui-Jun Jia, Jun-Ting Zhang

**Affiliations:** ^1^Department of Neurosurgery, Chinese People's Liberation Army General Hospital, Beijing, China; ^2^Department of Neurosurgery, Beijing Tian Tan Hospital, Capital Medical University, Beijing, China; ^3^China National Clinical Research Center for Neurological Diseases, Beijing, China; ^4^Center of Brain Tumor, Beijing Institute for Brain Disorders, Beijing, China; ^5^Beijing Key Laboratory of Brain Tumor, Beijing, China; ^6^Department of Radiotherapy, Beijing Fengtai You Anmen Hospital, Beijing, China

**Keywords:** invasiveness, radiosensitivity, microRNA-221/222, IOMM-Lee, dose rate, epithelial–mesenchymal transition-inducing transcription factors

## Abstract

The controversy of adjuvant radiotherapy of meningiomas is at least partially due to the insufficient understanding on meningioma cells' response to irradiation and the shortage of radiosensitivity-promotion methods. *MicroRNA-221* and *microRNA-222* were identified as critical regulators of radiosensitivity in several other tumors. However, their effect in meningiomas has yet to be confirmed. Therefore, the malignant meningioma IOMM-Lee cells were adopted, transfected with *microRNA-221/222* mimics or inhibitors, and irradiated with different dosages. The effects of radiation and *microRNA-221/222* were then assessed *in vitro* and *in vivo*. Radiation dose increases and *microRNA-221/222* downregulation synergistically inhibited cell proliferation and colony formation, prevented xenograft tumor progression, and promoted apoptosis, but antagonistically regulated cell invasiveness. Pairwise comparisons revealed that only high-dose radiations (6 and 8 Gy) can significantly promote cell invasiveness in comparison with unirradiated counterparts. Further comparisons exhibited that downregulating the *microRNA-221/222* expression can reverse this radiation-induced cell invasiveness to a level of untransfected and unirradiated cells only if cells were irradiated with no more than 6 Gy. In addition, this approach can promote IOMM-Lee's radiosensitivity. Meanwhile, we also detected that the dose rate of irradiation affects cell cycle distribution and cell apoptosis of IOMM-Lee. A high dose rate irradiation induces G0/G1 cell cycle arrest and apoptosis-promoting effect. Therefore, for malignant meningiomas, high-dose irradiation can facilitate cell invasiveness significantly. Downregulating the *microRNA-221/222* level can reverse the radiation-induced cell invasiveness while enhancing the apoptosis-promoting and proliferation-inhibiting effects of radiation and promoting cell radiosensitivity.

## Introduction

Meningiomas, one of the most common primary intracranial neoplasms, are classified into WHO grades I–III on the basis of local invasiveness and cellular features of atypia (1). Surgical resection is the primary treatment. As an important component of the therapeutic management of meningiomas, external beam radiotherapy aims to control tumor growth of surgically inaccessible tumors and in residual or recurrent lesions after surgery, ideally to achieve safe dose escalation and effective toxicity avoidance (e.g., necrosis of brain parenchyma, neurocognitive dysfunction, hypopituitarism, radiation-induced tumors, and malignant transformation) (2). However, radiotherapy has always been controversial, for instance, its necessity for WHO grade II lesions with different extents of resection (2–8), the optimal dosage (9–13), timing (7, 12, 14), etc. Thus, elucidation of how radiation exposure affects meningioma cells and exploration of the possible regulatory mechanism of radiosensitivity are indispensable for improved treatment.

MicroRNAs (miRs) are a family of endogenously synthesized small non-coding RNAs that regulate gene expression by influencing the protein translational machinery and/or inducing degeneration of target messenger RNAs (mRNAs) (15, 16). Genome-wide studies have demonstrated that miRNA genes are frequently located in cancer-associated genomic regions, indicating the potential roles of miRNAs in tumorigenesis (17). Previous studies on meningioma have suggested that several miRNAs participate in the regulation of cell proliferation (18–21), apoptosis (19, 22), invasiveness (19, 23), migration (19, 24), tumor recurrence (25–27), and histopathological progression (18, 19, 25, 27–29). However, no miRNAs have been verified to affect the radiosensitivity of meningiomas. *MiR-221* and *miR-222*, both located on the X chromosome with the same seed sequences, were confirmed to be involved in regulating the radiosensitivity of glioblastoma (30), gastric carcinoma (31), colorectal carcinoma (32, 33), and nasopharyngeal carcinoma (34). However, relevant research on the radiosensitivity of meningioma is lacking. In the present study, we aimed to reveal the effect of radiation on meningioma cells and the role of *miR-221/222* in regulating meningioma radiosensitivity.

## Materials and Methods

### Cells and Cell Culture

The meningioma cell line IOMM-Lee (ATCC Cat. No. CRL-3370, RRID: CVCL_5779) was kindly provided by Professor Jin-Hong Mei (Nanchang University, China) and was authenticated completely match with IOMM-Lee in the American Type Culture Collection (ATCC) short tandem repeat (STR) database without any cross-contamination of other human cell lines before and after this research. Cells were grown in Dulbecco's modified Eagle's medium (DMEM; HyClone, USA) supplemented with 10% fetal bovine serum at 37°C in a 5% CO_2_ atmosphere.

### Cell Transfection

The *miR-221/222* mimics and inhibitors were chemically synthesized by RiboBio Co., Ltd. (Guangzhou, China) and were transfected into IOMM-Lee cells with ribo*FECT*™ CP reagent according to the manufacturer's instructions. Scrambled oligonucleotides (GenePharma Co., Ltd., Shanghai, China) were also transfected as a negative control. The expression levels of *miR-221* and *miR-222* in transfected IOMM-Lee cells were identified by quantitative real-time PCR.

### Radiation Exposure

Irradiation was performed at room temperature in a linear accelerator (Varian600, Varian, USA) at a dose rate of 3.2 Gy/min (31, 33). Cells were plated into six-well plates and exposed to the specified dose (0, 2, 4, 6, and 8 Gy) of X-rays.

### Clonogenic Assay

A clonogenic assay was applied to determine the radiosensitivity of IOMM-Lee cells. A predetermined number of viable cells (1,000 cells for 0, 2, and 4 Gy; 2,000 cells for 6 and 8 Gy) were seeded in six-well culture plates and incubated at 37°C for 24 h. Next, the cells were irradiated with different doses and then incubated for 7 days to allow colony growth. Then, colonies were stained with crystal violet, and those containing 50 or more cells were counted. The plating efficiency was calculated by dividing the average number of counted colonies by the number of seeded cells. Survival fractions (SFs) were calculated by normalization to the plating efficiency of the respective unirradiated controls (32). After estimation of the SF at different radiation doses, the survival curve (log of SF vs. the radiation dose) was plotted, and the *D*_0_ value for each group was calculated using the following equation: SF = 1 – (1 – e^D/D0^)^n^(32). The D_0_ value, which represents the radiation dose required to reduce the SF from 100 to 37%, is considered a measure of the intrinsic radiosensitivity of cells (33). The sensitization enhancement ratio for each treated group was determined by the ratio of the D_0_ of the control group to that of the treated group (33).

### Cell Proliferation Assay

Cells were seeded into 96-well plates at a density of 2 × 10^3^ cells per well and cultured for 12 h. Cell proliferation was assessed using a Cell Counting Kit-8 assay (Fluorescence, Beijing, China) according to the manufacturer's instructions. Absorbance was measured at a wavelength of 450 nm on a Model 550 microplate reader (Bio-Rad Laboratories, Hercules, CA, USA).

### Cell Cycle and Apoptosis Analyses by Flow Cytometry

The effects of *miR-221/222* and irradiation on the cell cycle and apoptosis in IOMM-Lee cells were examined by flow cytometry. Pretreated IOMM-Lee cells in the log phase of growth were stained with Annexin V/fluorescein isothiocyanate (FITC) and propidium iodide (Beyotime, China). Cell cycle and apoptotic rate were examined with a fluorescence-activated cell-sorting (FACS) flow cytometer (BeamCyte, China), and the data were analyzed using CellQuest Software. The percentages of cells in G0/G1 phase and the apoptotic rate were measured by calculating the ratio of the number of corresponding cells and that of total cells. For each sample, 10,000 cells were measured.

### Invasion Assay

The invasive potential of the pretreated cells was evaluated by measuring the number of cells that invaded Matrigel-coated Transwell chambers. Prior to the experiment, Transwell inserts with 8-μm pores were coated with Matrigel and reconstituted with fresh medium for 2 h. Cells (1 × 10^5^/ml) were seeded into the upper chambers in 200 μl serum-free DMEM, while DMEM supplemented with 10% fetal bovine serum (700 μl) was placed in the lower chamber. After incubation for 48 h, cells that degraded the Matrigel and invaded the lower surface of the Matrigel-coated membrane were fixed with 70% ethanol, stained with hematoxylin, and counted in five random fields at a magnification × 200 under an optical microscope.

### Dual Luciferase Reporter Assay

The 3′-untranslated region (UTR) of phosphatase and tensin homolog (PTEN), which contains the predicted binding sites of *miR-221/222*, were cloned into the *Xho*I site of the psi-check2 reporter vector (Biomed, Beijing, China). For the luciferase reporter assays, IOMM-Lee cells were cultured in 24-well plates with three replicates, incubated for 24 h, and transfected with 500 ng of psi-check2-PTEN or psi-check2-control plasmids with/without 100 nM *miR-221* mimics or *miR-222* mimics using Lipofectamine 3000. Luciferase activity was measured 48 h after transfection using dual-luciferase reporter assay system (Promega, WI, USA) according to the manufacturer's procedures. Data were normalized by Firefly/Renilla luciferase activity.

### Western Blot Analysis

Protein of IOMM-Lee cells from each subgroup was extracted using radioimmunoprecipitation assay (RIPA) buffer (Beyotime, Shanghai, China). Their concentration was determined using a BCA Protein Assay Kit (Beyotime, Shanghai, China). Equal amounts of protein (5 μg) were then subjected to 10% sodium dodecyl sulfate–polyacrylamide gel electrophoresis (SDS-PAGE) followed by transfer of protein to polyvinylidene fluoride (PVDF) membranes (Millipore, Darmstadt, Germany). Membranes were subsequently blocked in Tris-buffered saline containing 0.1% Tween-20 and 5% skimmed milk powder and were incubated with primary antibodies against PTEN (1:1,000 dilution) and β-actin (1:4,000 dilution) (Cell Signaling Technology, MA, USA) overnight at 4°C. Horseradish peroxidase (HRP)-conjugated secondary antibodies for PTEN (1:2,000 dilution) and β-actin (1:4,000 dilution) (Cell Signaling Technology, MA, USA) were used afterward. The blots were detected using Pierce™ ECL Western Blotting Substrate (Thermo Fisher Scientific, Rockford, USA), and the membranes were developed using a ChemiDoc MP imaging system (Bio-Rad, Hercules, CA).

### *In vivo* Studies

All animal studies were conducted in accordance with an approved institutional animal care and use committee protocol of our hospital (202001014). IOMM-Lee cells (5 × 10^6^) were injected subcutaneously into the flank position of 5-week-old female BALB/c nude mice. When the tumors reached 5 mm in diameter, animals were randomly divided into 4 groups of 16 mice each and were, respectively treated with intratumoral injections of saline, scramble oligonucleotides, *miR-221/222-3p* agomirs, and *miR-221/222-3p* antagomirs (RiboBio, Guangzhou, China) every 4 days for a total of three doses (3 nmol/dose). Eight animals from each group were radiated with two doses of 4 Gy during the intervals between injections. The entire mouse body except the tumor area was covered with lead sheets to avoid exposure to radiation during treatments. Vernier caliper was used to measure the length and width of tumors on alternate days, and tumor volumes were calculated as π/6 × (length × width^2^). Regression in subcutaneous tumor growth was followed, and mice were euthanized when tumor rupture and hemorrhage were observed in unradiated-control group. Immediately after the removal of the tumors, half of each tumor was stored in liquid nitrogen for the subsequent quantification of *miR-221* and *miR-222* by using quantitative real-time PCR (qRT-PCR); the other half was fixed in buffered formaldehyde and was sectioned and subjected to the later H&E and immunohistochemical staining for PTEN.

### Statistical Analysis

The abovementioned experiments were performed at least in triplicate, and data are presented as the mean ± standard deviation. The effects of *miR-221/222* expression level and radiation dose on IOMM-Lee cells *in vitro* and *in vivo* were tested with two-way analysis of variance. Simple effect and pairwise comparisons with Bonferroni posttest were performed if the interaction between the factors appeared significant; otherwise, main effect and multiple comparisons with Bonferroni posttest were performed. Further comparisons of invasive cell numbers between the inhibitor group and the control/scramble group exposed to different radiation doses were analyzed by independent-sample *t*-tests. Multiple comparisons of xenograft tumor volumes between different treatment groups were analyzed by ANOVA with Bonferroni posttest. All *P* values are two-sided, and significance was defined using a threshold of 0.05. Statistical analyses were performed with SPSS version 19.0 (IBM Corp. Armonk, New York, USA).

## Results

### Modulation of *miR-221/222* Expression in IOMM-Lee Cell Line

**Figure 1:**
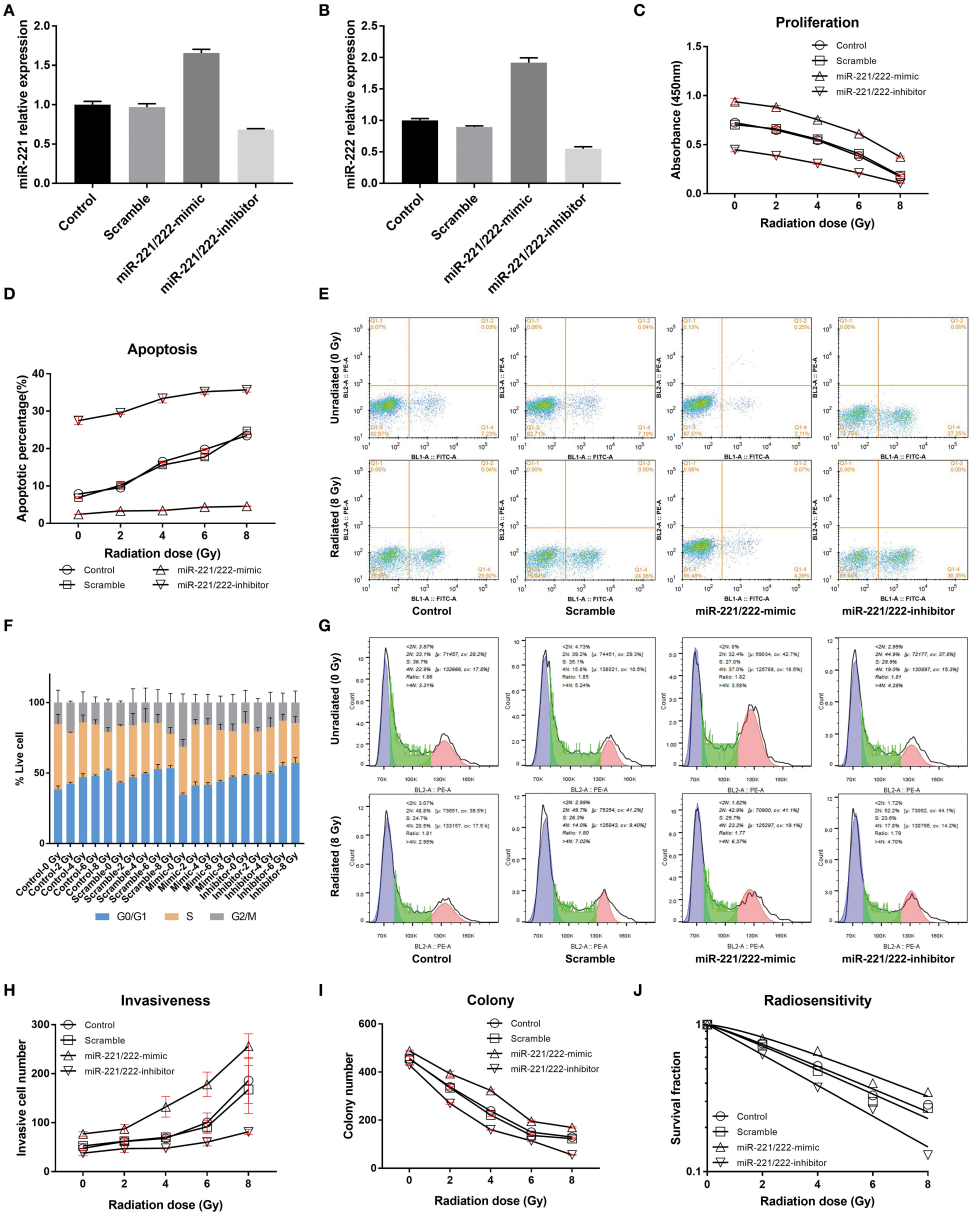
The effects of the expression of *miR-221/22*2 and radiation dose on IOMM-Lee cells *in vitro*. **(A,B)** Present the relative expressions of *miR-221* and *miR-222* in different groups after transfection, respectively. **(C)** Shows that increasing the radiation dose and downregulating *miR-221/222* expression level can synergistically inhibit the proliferation of IOMM-Lee cells, while **(D,E)** show their synergistical promotion on cell apoptosis. **(F,G)** Exhibit that both increasing the radiation dose and downregulating *miR-221/222* expression level can separately increase the sub-G0/G1 population and induce G0/G1 cell cycle arrest. **(H,I)** Revealed that increasing the radiation dose and downregulating *miR-221/222* expression level can antagonistically regulate the cell invasiveness, while synergistically inhibit the colony formation, respectively. Furthermore, **(J)** exhibits that downregulating the *miR-221/222* expression enhances the radiosensitivity of IOMM-Lee cells. The abovementioned experiments were performed at least in triplicate.

IOMM-Lee cells were transfected with *miR-221/222* mimics or inhibitors. qRT-PCR revealed that no significant difference in *miR-221* and *miR-222* expression between the control and the scramble group (*miR-221*: *P* = 0.7640, *miR-222*: *P* = 0.0856). Compared with that in either the control or the scramble group, the expression of *miR-221* and *miR-222* increased significantly in the *miR-221/222*-mimic group (*miR-221*: vs. control, *P* < 0.0001; vs. scramble, *P* < 0.0001; *miR-222*: vs. control, *P* < 0.0001; vs. scramble, *P* < 0.0001), while it decreased significantly in the *miR-221/222*-inhibitor group (*miR-221*: vs. control, *P* < 0.0001; vs. scramble, *P* < 0.0001; *miR-222*: vs. control, *P* < 0.0001; vs. scramble, *P* < 0.0001) (Figures 1A,B).

### Radiation Dose and Expression Level of *miR-221/222* Synergistically Modulate IOMM-Lee Cell Proliferation, Apoptosis, and Cell Cycle Distribution

With an increase in radiation dose or a decrease in *miR-221/222* expression level, the absorbance and colony number of IOMM-Lee cells decreased gradually, while apoptotic percentage and G0/G1 phase percentage increased; however, their invasive cell number increased as the radiation dose increased and decreased as the *miR-221/222* expression level decreased (Table 1, Figure 1). Two-way ANOVA revealed significant simple effects of radiation dose and *miR-221/222* expression level and their significant interactions in the proliferation, colony formation, apoptosis, and invasiveness of IOMM-Lee cells, while it exhibited their significant main effects in the sub-G0/G1 population, yet without significant interactions (Table 2).

**Table 1:** Descriptive statistics of various assays of IOMM-Lee cells.

**Different assays**	**Number**	**Group (Mean** **±** **Standard deviation)**			
		**Control**	**Scramble**	**Mimic**	**Inhibitor**
**Absorbance (450 nm)**					
0 Gy	3	0.724 ± 0.008	0.705 ± 0.011	0.938 ± 0.032	0.450 ± 0.023
2 Gy	3	0.649 ± 0.029	0.660 ± 0.023	0.883 ± 0.016	0.387 ± 0.006
4 Gy	3	0.545 ± 0.007	0.555 ± 0.024	0.754 ± 0.020	0.307 ± 0.009
6 Gy	3	0.383 ± 0.016	0.406 ± 0.022	0.612 ± 0.005	0.210 ± 0.008
8 Gy	3	0.174 ± 0.021	0.183 ± 0.010	0.374 ± 0.011	0.108 ± 0.006
**Colony number**					
0 Gy	3	454.333 ± 8.021	456.333 ± 8.021	487.667 ± 3.786	429.000 ± 2.000
2 Gy	3	338.333 ± 9.713	334.000 ± 4.583	393.000 ± 10.536	269.000 ± 3.606
4 Gy	3	238.000 ± 3.000	221.000 ± 5.568	323.333 ± 5.686	160.333 ± 2.082
6 Gy^‡^	3	150.000 ± 3.606	136.667 ± 2.517	195.000 ± 5.000	114.000 ± 3.000
8 Gy^‡^	3	129.667 ± 4.933	123.333 ± 4.163	169.667 ± 3.055	55.667 ± 1.528
**Apoptotic percentage (%)**					
0 Gy	3	7.850 ± 0.684	6.897 ± 0.302	2.407 ± 0.057	27.473 ± 1.033
2 Gy	3	9.587 ± 0.023	10.190 ± 0.710	3.313 ± 0.201	29.540 ± 0.376
4 Gy	3	16.490 ± 0.154	15.607 ± 0.580	3.457 ± 0.068	33.413 ± 1.196
6 Gy	3	19.743 ± 0.321	17.750 ± 0.700	4.340 ± 0.147	35.213 ± 0.671
8 Gy	3	23.503 ± 0.588	24.743 ± 0.337	4.570 ± 0.101	35.717 ± 0.611
**Cell cycle distribution (%)**^¶^					
**G0/G1 phase**					
0 Gy	3	38.412 ± 2.400	43.192 ± 0.614	34.748 ± 1.102	48.659 ± 0.316
2 Gy	3	42.388 ± 1.074	47.115 ± 1.426	41.311 ± 2.411	48.919 ± 1.052
4 Gy	3	46.981 ± 2.555	49.741 ± 0.699	41.694 ± 1.472	50.139 ± 1.066
6 Gy	3	47.949 ± 0.743	52.801 ± 3.184	43.921 ± 0.920	55.094 ± 2.479
8 Gy	3	51.974 ± 0.791	53.391 ± 1.913	47.220 ± 0.725	57.241 ± 3.615
**S phase**					
0 Gy	3	46.262 ± 6.932	36.459 ± 0.114	39.013 ± 5.147	36.517 ± 3.209
2 Gy	3	27.145 ± 3.136	40.027 ± 0.962	36.787 ± 8.346	35.985 ± 9.891
4 Gy	3	32.658 ± 5.989	24.266 ± 4.618	33.796 ± 5.214	43.053 ± 3.468
6 Gy	3	42.525 ± 4.232	36.462 ± 4.193	32.500 ± 5.032	36.545 ± 8.268
8 Gy	3	30.511 ± 2.680	32.301 ± 6.639	31.983 ± 4.564	28.289 ± 4.606
**G2/M phase**					
0 Gy	3	15.326 ± 8.769	21.153 ± 0.962	14.006 ± 7.538	15.534 ± 3.943
2 Gy	3	20.881 ± 2.394	16.782 ± 1.038	16.099 ± 9.762	14.274 ± 10.200
4 Gy	3	14.540 ± 9.162	22.343 ± 6.500	31.456 ± 6.072	15.637 ± 5.816
6 Gy	3	15.781 ± 5.692	19.617 ± 4.586	20.280 ± 5.735	14.796 ± 8.574
8 Gy	3	20.570 ± 2.853	17.560 ± 7.330	12.924 ± 6.696	14.470 ± 8.155
**Invasive cell number**					
0 Gy	5	47.800 ± 1.643	52.600 ± 4.980	77.000 ± 4.583	37.800 ± 5.215
2 Gy	5	61.600 ± 4.827	62.000 ± 6.403	87.200 ± 9.039	47.000 ± 7.517
4 Gy	5	68.200 ± 3.194	70.200 ± 4.658	132.400 ± 20.959	47.800 ± 1.304
6 Gy	5	100.400 ± 19.424	91.600 ± 13.334	178.200 ± 25.024	60.400 ± 7.893
8 Gy	5	186.200 ± 47.108	167.600 ± 48.993	256.000 ± 25.318	81.400 ± 5.320

‡ *These results were normalized as the colony numbers per 1000 seeded cells*.

¶ *These results, after the exclusion of “**<** 2N” and “> 4N” parts, were normalized by using the geometric proportion method to achieve the sum of persentages of G0/G1, S, G2/M phases of each subgroup is 100%*.

**Table 2:** Radiation dose and expression level of miR-221/222 co-modulate IOMM-Lee cells.

**Different assays**	**Source**	**df**	***F***	***P***	**Partial eta squared**
**Absorbance (450 nm)**	Group	3	1458.879	<0.0001*	0.991
	Radiation dose	4	1567.872	<0.0001*	0.994
	Group × Radiation dose	12	19.320	<0.0001*	0.853
	Error	40			
**Colony number**	Group	3	1032.021	<0.0001*	0.987
	Radiation dose	4	8088.989	<0.0001*	0.999
	Group × Radiation dose	12	33.749	<0.0001*	0.910
	Error	40			
**Apoptotic percentage**	Group	3	2089.699	<0.0001*	0.998
	Radiation dose	4	238.783	<0.0001*	0.988
	Group × Radiation dose	12	26.278	0.0038*	0.963
	Error	40			
**Cell cycle distribution**					
G0/G1 phase	Group	3	93.727	<0.0001*	0.875
	Radiation dose	4	71.541	<0.0001*	0.877
	Group × Radiation dose	12	1.870	0.0691	0.359
	Error	40			
S phase	Group	3	0.511	0.6767	0.037
	Radiation dose	4	4.643	0.0036*	0.317
	Group × Radiation dose	12	3.256	0.0024*	0.494
	Error	40			
G2/M phase	Group	3	1.406	0.2551	0.095
	Radiation dose	4	0.982	0.4283	0.089
	Group × Radiation dose	12	1.297	0.2584	0.280
	Error	40			
**Invasive cell number**	Group	3	97.670	<0.0001*	0.786
	Radiation dose	4	124.624	<0.0001*	0.862
	Group × Radiation dose	12	8.406	<0.0001*	0.558
	Error	80			

* *Indicates statistical significance, and relevant P values are emphasized in bold*.

Increasing radiation dose and downregulating *miR-221/222* expression have synergistic effects on inhibiting proliferation and promoting apoptosis of IOMM-Lee cells (Tables 1, 2). Significant decrease in cell absorbance and colony number appears at each step-up of irradiation dose or each fall of the *miR-221/222* expression (Tables 3, 5 and Figures 1C,I), indicating that the proliferation-inhibiting effect of radiation can be significantly enhanced by downregulating *miR-221/222* expression.

**Table 3:** Pairwise comparisons between IOMM-Lee cells irradiated with different dosages in various assays.

**Pairwise comparisons**	**Increased dosage**	**Control**			**Scramble**			**Mimic**			**Inhibitor**		
		***P***	**95% CI**		***P***	**95% CI**		***P***	**95% CI**		***P***	**95% CI**	
**Absorbance (450 nm)**													
0 Gy vs. 2 Gy	2 Gy	<0.0001*	0.033	0.118	0.0313*	0.002	0.087	0.0036*	0.013	0.098	0.0007*	0.021	0.106
2 Gy vs. 4 Gy	2 Gy	<0.0001*	0.062	0.147	<0.0001*	0.063	0.148	<0.0001*	0.086	0.171	<0.0001*	0.038	0.122
4 Gy vs. 6 Gy	2 Gy	<0.0001*	0.120	0.204	<0.0001*	0.106	0.191	<0.0001*	0.100	0.185	<0.0001*	0.054	0.139
6 Gy vs. 8 Gy	2 Gy	<0.0001*	0.167	0.251	<0.0001*	0.329	0.414	<0.0001*	0.195	0.280	<0.0001*	0.060	0.145
0 Gy vs. 4 Gy	4 Gy	<0.0001*	0.137	0.222	<0.0001*	0.108	0.192	<0.0001*	0.142	0.226	<0.0001*	0.101	0.186
2 Gy vs. 6 Gy	4 Gy	<0.0001*	0.224	0.309	<0.0001*	0.211	0.296	<0.0001*	0.229	0.314	<0.0001*	0.134	0.219
4 Gy vs. 8 Gy	4 Gy	<0.0001*	0.329	0.413	<0.0001*	0.181	0.266	<0.0001*	0.338	0.423	<0.0001*	0.157	0.241
0 Gy vs. 6 Gy	6 Gy	<0.0001*	0.299	0.384	<0.0001*	0.256	0.341	<0.0001*	0.284	0.369	<0.0001*	0.198	0.282
2 Gy vs. 8 Gy	6 Gy	<0.0001*	0.433	0.518	<0.0001*	0.435	0.519	<0.0001*	0.467	0.551	<0.0001*	0.237	0.321
0 Gy vs. 8 Gy	8 Gy	<0.0001*	0.508	0.593	<0.0001*	0.479	0.564	<0.0001*	0.522	0.607	<0.0001*	0.300	0.385
**Colony number**													
0 Gy vs. 2 Gy	2 Gy	<0.0001*	103.053	128.947	<0.0001*	109.386	135.281	<0.0001*	81.719	107.614	<0.0001*	147.053	172.947
2 Gy vs. 4 Gy	2 Gy	<0.0001*	87.386	113.281	<0.0001*	100.053	125.947	<0.0001*	56.719	82.614	<0.0001*	95.719	121.614
4 Gy vs. 6 Gy	2 Gy	<0.0001*	75.053	100.947	<0.0001*	71.386	97.281	<0.0001*	115.386	141.281	<0.0001*	33.386	59.281
6 Gy vs. 8 Gy	2 Gy	0.0003*	7.386	33.281	0.0394*	0.386	26.281	<0.0001*	12.386	38.281	<0.0001*	45.386	71.281
0 Gy vs. 4 Gy	4 Gy	<0.0001*	203.386	229.281	<0.0001*	222.386	248.281	<0.0001*	151.386	177.281	<0.0001*	255.719	281.614
2 Gy vs. 6 Gy	4 Gy	<0.0001*	175.386	201.281	<0.0001*	184.386	210.281	<0.0001*	185.053	210.947	<0.0001*	142.053	167.947
4 Gy vs. 8 Gy	4 Gy	<0.0001*	95.386	121.281	<0.0001*	84.719	110.614	<0.0001*	140.719	166.614	<0.0001*	91.719	117.614
0 Gy vs. 6 Gy	6 Gy	<0.0001*	291.386	317.281	<0.0001*	306.719	332.614	<0.0001*	279.719	305.614	<0.0001*	302.053	327.947
2 Gy vs. 8 Gy	6 Gy	<0.0001*	195.719	221.614	<0.0001*	197.719	223.614	<0.0001*	210.386	236.281	<0.0001*	200.386	226.281
0 Gy vs. 8 Gy	8 Gy	<0.0001*	311.719	337.614	<0.0001*	320.053	345.947	<0.0001*	305.053	330.947	<0.0001*	360.386	386.281
**Apoptotic percentage**													
0 Gy vs. 2 Gy	2 Gy	0.0038*	−3.068	0.406	<0.0001*	−4.624	−1.962	0.4969	−2.238	0.424	0.0004*	−3.398	−0.736
2 Gy vs. 4 Gy	2 Gy	<0.0001*	−8.234	−5.572	<0.0001*	−6.748	−4.086	1.0000	−1.474	1.188	<0.0001*	−5.204	−2.542
4 Gy vs. 6 Gy	2 Gy	<0.0001*	−4.584	−1.922	0.0002*	−3.474	−0.812	0.5557	−2.214	0.448	0.0025*	−3.131	−0.469
6 Gy vs. 8 Gy	2 Gy	<0.0001*	−5.091	−2.429	<0.0001*	−8.324	−5.662	1.0000	−1.561	1.101	1.0000	−1.834	0.828
0 Gy vs. 4 Gy	4 Gy	<0.0001*	−9.971	−7.309	<0.0001*	−10.041	−7.379	0.2414	−2.381	0.281	<0.0001*	−7.271	−4.609
2 Gy vs. 6 Gy	4 Gy	<0.0001*	−11.488	−8.826	<0.0001*	−8.891	−6.229	0.2726	−2.358	0.304	<0.0001*	−7.004	−4.342
4 Gy vs. 8 Gy	4 Gy	<0.0001*	−8.344	−5.682	<0.0001*	−10.468	−7.806	0.1723	−2.444	0.218	0.0001*	−3.634	−0.972
0 Gy vs. 6 Gy	6 Gy	<0.0001*	−13.224	−10.562	<0.0001*	−12.184	−9.522	0.0010*	−3.264	−0.602	<0.0001*	−9.071	−6.409
2 Gy vs. 8 Gy	6 Gy	<0.0001*	−15.248	−12.586	<0.0001*	−15.884	−13.222	0.0773	−2.588	0.074	<0.0001*	−7.508	−4.846
0 Gy vs. 8 Gy	8 Gy	<0.0001*	−16.984	−14.322	<0.0001*	−19.178	−16.516	0.0002*	−3.494	−0.832	<0.0001*	−9.574	−6.912
**Cell cycle distribution (S phase)**													
0 Gy vs. 2 Gy	2 Gy	0.0009*	6.031	32.204	1.0000	−16.654	9.519	1.0000	−10.860	15.313	1.0000	−12.555	13.618
2 Gy vs. 4 Gy	2 Gy	1.0000	−18.600	7.573	0.0092*	2.674	28.848	1.0000	−10.096	16.077	1.0000	−20.154	6.019
4 Gy vs. 6 Gy	2 Gy	0.3071	−22.953	3.220	0.0848	−25.283	0.890	1.0000	−11.790	14.383	1.0000	−6.579	19.594
6 Gy vs. 8 Gy	2 Gy	0.0943	−1.073	25.100	1.0000	−8.925	17.248	1.0000	−12.569	13.604	0.6819	−4.831	21.343
0 Gy vs. 4 Gy	4 Gy	0.0365*	0.517	26.690	0.0849	−0.893	25.280	1.0000	−7.870	18.303	1.0000	−19.623	6.55
2 Gy vs. 6 Gy	4 Gy	0.0118*	−28.467	−2.294	1.0000	−9.522	16.651	1.0000	−8.800	17.373	1.0000	−13.646	12.527
4 Gy vs. 8 Gy	4 Gy	1.0000	−10.940	15.234	0.7559	−21.122	5.052	1.0000	−11.273	14.900	0.0176*	1.677	27.85
0 Gy vs. 6 Gy	6 Gy	1.0000	−9.349	16.824	1.0000	−13.090	13.083	1.0000	−6.574	19.600	1.0000	−13.115	13.058
2 Gy vs. 8 Gy	6 Gy	1.0000	−16.453	9.720	0.8707	−5.361	20.813	1.0000	−8.283	17.890	0.8825	−5.39	20.783
0 Gy vs. 8 Gy	8 Gy	0.0093*	2.664	28.838	1.0000	−8.928	17.245	1.0000	−6.056	20.117	0.6910	−4.859	21.314
**Invasive cell number**													
0 Gy vs. 2 Gy	2 Gy	1.0000	−48.644	21.044	1.0000	−44.244	25.444	1.0000	−45.044	24.644	1.0000	−44.044	25.644
2 Gy vs. 4 Gy	2 Gy	1.0000	−41.444	28.244	1.0000	−43.044	26.644	0.0034*	−80.044	−10.356	1.0000	−35.644	34.044
4 Gy vs. 6 Gy	2 Gy	0.0924	−67.044	2.644	0.8002	−56.244	13.444	0.0029*	−80.644	−10.956	1.0000	−47.444	22.244
6 Gy vs. 8 Gy	2 Gy	<0.0001*	−120.644	−50.956	<0.0001*	−110.844	−41.156	<0.0001*	−112.644	−42.956	0.8572	−55.844	13.844
0 Gy vs. 4 Gy	4 Gy	0.9488	−55.244	14.444	1.0000	−52.444	17.244	0.0002*	−90.244	−20.556	1.0000	−44.844	24.844
2 Gy vs. 6 Gy	4 Gy	0.0188*	−73.644	−3.956	0.1636	−64.444	5.244	<0.0001*	−125.844	−56.156	1.0000	−48.244	21.444
4 Gy vs. 8 Gy	4 Gy	<0.0001*	−152.844	−83.156	<0.0001*	−132.244	−62.556	<0.0001*	−158.444	−88.756	0.0670	−68.444	1.244
0 Gy vs. 6 Gy	6 Gy	0.0004*	−87.444	−17.756	0.0179*	−73.844	−4.156	<0.0001*	−136.044	−66.356	0.6479	−57.444	12.244
2 Gy vs. 8 Gy	6 Gy	<0.0001*	−159.444	−89.756	<0.0001*	−140.444	−70.756	<0.0001*	−203.644	−133.956	0.0556	−69.244	0.444
0 Gy vs. 8 Gy	8 Gy	<0.0001*	−173.244	−103.556	<0.0001*	−149.844	−80.156	<0.0001*	−213.844	−144.156	0.0053*	−78.444	−8.756

* *Indicates statistical significance, and relevant P values are emphasized in bold*.

As to the apoptosis of IOMM-Lee cells, pairwise comparisons of different groups revealed that, by irradiating with the same dosage, the apoptosis rate was significantly increased with downregulation of *miR-221/222* expression (**Table 5** and Figures 1D,E). Meanwhile, (1) the radiation dose that initially significantly promoted cell apoptosis was much higher in the *miR-221/222*-mimic group (6 Gy) than in the other groups (2 Gy) compared to their respective unirradiated cells; (2) within irradiated IOMM-Lee cells, the significant increase in apoptotic rate caused by each step-up of irradiation dose, which can be seen in cells with regular or decreased *miR-221/222* expression, was not observed as the expression of *miR-221/222* promoted; (3) in the comparisons between two irradiated subgroups with an incremental gradient of 4 or 6 Gy, the apoptosis rate increased significantly in the control, scramble, and *miR-221/222*-inhibitor group, whereas no significant differences were detected in the *miR-221/222*-mimic group (Table 3 and Figures 1D,E). These findings, from different perspectives, suggest that the apoptosis-promoting effect of radiation can be significantly enhanced by downregulating *miR-221/222* expression in IOMM-Lee cells.

Further analysis of cell cycle distribution exhibits that the sub-G0/G1 population was positively correlated with radiation dose but negatively correlated with *miR-221/222* expression (Table 4, Figures 1F,G). No corresponding effects on the sub-G2/M population were found. Although a significant effect of radiation dose on the S phase population was presented with an interaction with the *miR-221/222* expression level (Table 2), no obvious radiation dose-dependent trend was explored in pairwise comparisons (Table 3).

**Table 4:** Multiple comparisons of the persentage of G0/G1 phase between different IOMM-Lee cell groups.

**Multiple comparisons**	***P***	**95% CI**	
0 Gy vs. 2 Gy	0.0001*	−5.841	−1.520
2 Gy vs. 4 Gy	0.0423*	−4.366	−0.045
4 Gy vs. 6 Gy	0.0041*	−4.963	−0.642
6 Gy vs. 8 Gy	0.0130*	−4.676	−0.355
0 Gy vs. 4 Gy	<0.0001*	−8.046	−3.726
2 Gy vs. 6 Gy	<0.0001*	−7.169	−2.848
4 Gy vs. 8 Gy	<0.0001*	−7.478	−3.157
0 Gy vs. 6 Gy	<0.0001*	−10.849	−6.528
2 Gy vs. 8 Gy	<0.0001*	−9.684	−5.363
0 Gy vs. 8 Gy	<0.0001*	−13.364	−9.043
Control vs. Scramble	<0.0001*	−5.512	−1.902
Mimic vs. Control	<0.0001*	−5.567	−1.957
Mimic vs. Scramble	<0.0001*	−9.274	−5.664
Inhibitor vs. Control	<0.0001*	4.664	8.275
Inhibitor vs. Scramble	0.0008*	0.957	4.568
Mimic vs. Inhibitor	<0.0001*	−12.037	−8.426

* *Indicates statistical significance, and relevant P values are emphasized in bold*.

### Radiation Dose and Expression Level of *miR-221/222* Antagonistically Modulate IOMM-Lee Cell Invasion

Increased radiation dose and downregulated *miR-221/222* have antagonistic effects on cell invasiveness (Tables 1, 2). Pairwise comparison analysis revealed that (1) invasive cell number at 8 Gy was significantly higher than that at lower radiation doses in the control or the scramble group, while invasive cell number at 6 Gy was only significantly higher than that of the unirradiated/2 Gy-irradiated control groups and unirradiated scramble group, respectively; (2) in the *miR-221/222*-mimic group, the invasive cell number for cells irradiated with a dose no lower than 4 Gy was significantly higher than that at lower radiation doses. However, in the *miR-221/222*-inhibitor group, the invasive cell number was significantly increased only in the comparison of 0 vs. 8 Gy (Table 3 and Figure 1H); (3) the expression level of *miR-221/222* had no significant effect on cell invasiveness at a low radiation dose (≤2 Gy) compared to that of the control or the scramble group. Only at high radiation doses did the high expression of *miR-221/222* exhibit a significant invasion-promoting effect (≥4 Gy), while the low expression of *miR-221/222* presented a significant invasion-inhibiting effect (≥6 Gy) (Table 5 and Figure 1H).

**Table 5:** Pairwise comparisons between IOMM-Lee cells with different miR−221/222 expression levels in various assays.

**Pairwise comparisons**	**0 Gy**			**2 Gy**			**4 Gy**			**6 Gy**			**8 Gy**		
	***P***	**95% CI**		***P***	**95% CI**		***P***	**95% CI**		***P***	**95% CI**		***P***	**95% CI**	
**Absorbance (450 nm)**															
Control vs. Scramble	1.0000	−0.020	0.059	1.0000	−0.050	0.028	1.0000	−0.049	0.029	0.6205	−0.063	0.016	1.0000	−0.049	0.030
Mimic vs. Control	<0.0001*	0.175	0.253	<0.0001*	0.195	0.273	<0.0001*	0.170	0.249	<0.0001*	0.190	0.268	<0.0001*	0.161	0.240
Mimic vs. Scramble	<0.0001*	0.194	0.273	<0.0001*	0.184	0.262	<0.0001*	0.160	0.239	<0.0001*	0.166	0.245	<0.0001*	0.152	0.230
Inhibitor vs. Control	<0.0001*	−0.314	−0.235	<0.0001*	−0.302	−0.223	<0.0001*	−0.277	−0.199	<0.0001*	−0.212	−0.133	0.0002*	−0.105	−0.027
Inhibitor vs. Scramble	<0.0001*	−0.294	−0.215	<0.0001*	−0.313	−0.234	<0.0001*	−0.287	−0.209	<0.0001*	−0.236	−0.157	<0.0001*	−0.115	−0.036
Mimic vs. Inhibitor	<0.0001*	0.449	0.528	<0.0001*	0.457	0.536	<0.0001*	0.408	0.487	<0.0001*	0.362	0.441	<0.0001*	0.227	0.306
**Colony number**															
Control vs. Scramble	1.0000	−14.096	10.096	1.0000	−7.763	16.430	0.0021*	4.904	29.096	0.0237*	1.237	25.430	0.9235	−5.763	18.430
Mimic vs. Control	<0.0001*	21.237	45.430	<0.0001*	42.570	66.763	<0.0001*	73.237	97.430	<0.0001*	32.904	57.096	<0.0001*	27.904	52.096
Mimic vs. Scramble	<0.0001*	19.237	43.430	<0.0001*	46.904	71.096	<0.0001*	90.237	114.430	<0.0001*	46.237	70.430	<0.0001*	34.237	58.430
Inhibitor vs. Control	<0.0001*	−37.430	−13.237	<0.0001*	−81.430	−57.237	<0.0001*	−89.763	−65.570	<0.0001*	−48.096	−23.904	<0.0001*	−86.096	−61.904
Inhibitor vs. Scramble	<0.0001*	−39.430	−15.237	<0.0001*	−77.096	−52.904	<0.0001*	−72.763	−48.570	<0.0001*	−34.763	−10.570	<0.0001*	−79.763	−55.570
Mimic vs. Inhibitor	<0.0001*	46.570	70.763	<0.0001*	111.904	136.096	<0.0001*	150.904	175.096	<0.0001*	68.904	93.096	<0.0001*	101.904	126.096
**Apoptotic percentage**															
Control vs. Scramble	0.2373	−0.290	2.197	1.0000	−1.847	0.640	0.3334	−0.360	2.127	0.0004*	0.750	3.237	0.0510	−2.484	0.004
Mimic vs. Control	<0.0001*	−6.687	−4.200	<0.0001*	−7.517	−5.030	<0.0001*	−14.277	−11.790	<0.0001*	−16.647	−14.160	<0.0001*	−20.177	−17.690
Mimic vs. Scramble	<0.0001*	−5.734	−3.246	<0.0001*	−8.120	−5.633	<0.0001*	−13.394	−10.906	<0.0001*	−14.654	−12.166	<0.0001*	−21.417	−18.930
Inhibitor vs. Control	<0.0001*	18.380	20.867	<0.0001*	18.710	21.197	<0.0001*	15.680	18.167	<0.0001*	14.226	16.714	<0.0001*	10.970	13.457
Inhibitor vs. Scramble	<0.0001*	19.333	21.820	<0.0001*	18.106	20.594	<0.0001*	16.563	19.050	<0.0001*	16.220	18.707	<0.0001*	9.730	12.217
Mimic vs. Inhibitor	<0.0001*	−26.310	−23.823	<0.0001*	−27.470	−24.983	<0.0001*	−31.200	−28.713	<0.0001*	−32.117	−29.630	<0.0001*	−32.390	−29.903
**Invasive cell number**															
Control vs. Scramble	1.0000	−37.453	27.853	1.0000	−33.053	32.253	1.0000	−34.653	30.653	1.0000	−23.853	41.453	0.7634	−14.053	51.253
Mimic vs. Control	0.1069	−3.453	61.853	0.2221	−7.053	58.253	<0.0001*	31.547	96.853	<0.0001*	45.147	110.453	<0.0001*	37.147	102.453
Mimic vs. Scramble	0.2793	−8.253	57.053	0.2399	−7.453	57.853	<0.0001*	29.547	94.853	<0.0001*	53.947	119.253	<0.0001*	55.747	121.053
Inhibitor vs. Control	1.0000	−42.653	22.653	1.0000	−47.253	18.053	0.5693	−53.053	12.253	0.0083*	−72.653	−7.347	<0.0001*	−137.453	−72.147
Inhibitor vs. Scramble	1.0000	−47.453	17.853	1.0000	−47.653	17.653	0.4029	−55.053	10.253	0.0693	−63.853	1.453	<0.0001*	−118.853	−53.547
Mimic vs. Inhibitor	0.0102*	6.547	71.853	0.0079*	7.547	72.853	<0.0001*	51.947	117.253	<0.0001*	85.147	150.453	<0.0001*	141.947	207.253

* *Indicates statistical significance, and relevant P values are emphasized in bold*.

By further comparing the invasive cell numbers of the inhibitor group and the control/scramble group exposed to different irradiation doses, it was revealed that although failed to completely reverse the 8-Gy-promoted invasiveness to a low-dose radiation-induced level, downregulation of *miR-221/222* expression can completely reverse the 6-Gy-induced cell invasiveness to a level, which is without significant increase compared with that of the low-dose-irradiated control groups or unirradiated/low-dose-irradiated scramble groups (Tables 1, 6).

**Table 6:** Comparisons between the invasive cell numbers of the miR-221/222-inhibitor and the control/scramble group exposed to different radiation doses.

	**0 Gy-Inhibitor**			**2 Gy-Inhibitor**			**4 Gy-Inhibitor**			**6 Gy-Inhibitor**			**8 Gy-Inhibitor**		
	***P***	**95% CI**		***P***	**95% CI**		***P***	**95% CI**		***P***	**95% CI**		***P***	**95% CI**	
0 Gy-Control	0.0035*	4.361	15.639	0.8266	-8.434	10.034	1.0000	-2.163	2.163	0.0219*	-22.304	−2.896	<0.0001*	−39.342	−27.858
2 Gy-Control				0.0065*	5.388	23.812	0.0003*	8.644	18.956	0.7792	-8.341	10.741	0.0003*	−27.208	−12.392
4 Gy-Control							<0.0001*	16.842	23.958	0.0929	-1.837	17.437	0.0014*	−19.599	−6.801
6 Gy-Control										0.0070*	16.284	63.716	0.0935	−4.777	42.777
8 Gy-Control													0.0011*	55.909	153.691
0 Gy-Scramble	0.0018*	7.363	22.237	0.2023	-3.699	14.899	0.0706	-0.509	10.109	0.0986	-17.425	1.825	<0.0001*	−36.315	−21.285
2 Gy-Scramble				0.0094*	4.817	25.183	0.0013*	7.461	20.939	0.7339	-8.882	12.082	0.0008*	−27.985	−10.815
4 Gy-Scramble							0.0002*	16.700	28.100	0.0438*	0.348	19.252	0.0076*	−18.492	−3.908
6 Gy-Scramble										0.0020*	15.220	47.180	0.1508	−4.605	25.005
8 Gy-Scramble													0.0166*	25.562	146.838

* *Indicates statistical significance, and relevant P values are emphasized in bold*.

### Downregulation of *miR-221/222* Expression Promotes Radiosensitivity of IOMM-Lee Cells

The effect of genetic manipulation of *miR-221/222* on radiosensitivity of IOMM-Lee cells was investigated using a clonogenic assay. The D_0_ value of the control, scramble, *miR-221/222*-mimic, and *miR-221/222*-inhibitor groups are 5.4242, 5.0970, 5.6025, and 4.1296 Gy, respectively. The sensitization enhancement ratio (SER) was 1.0642, 0.9682, and 1.3135 for the scramble, *miR-221/222*-mimic, and *miR-221/222*-inhibitor groups, respectively (Table 7, Figure 1J). These results revealed a negative correlation between the SER and the *miR-221/222* expression, which provides strong evidence that downregulation of *miR-221/222* expression can promote the radiosensitivity of IOMM-Lee cells.

**Table 7:** Impact of miRNA-221/222 expression on IOMM-Lee cell radiosensitivity.

**Group**	**D_**0**_**	**Dq**	**SF_**2**_**	**SER**
Control + irradiation	5.4242	0.6797	0.7364	
Scramble + irradiation	5.0970	0.5713	0.7160	1.0642
miRNA-221/222-mimic + irradiation	5.6025	2.0211	0.8220	0.9682
miRNA-221/222-inhibitor + irradiation	4.1296	0.1000	0.6250	1.3135

*D_0_ is a dose that reduces the survival fraction from 100% to 37%; Dq, quasi-threshold dose; SF2, survival fraction at 2 Gy; SER, the sensitization enhancement ratio*.

### PTEN Is a Target Gene of the *miR-221/222* Cluster

**Figure 2:**
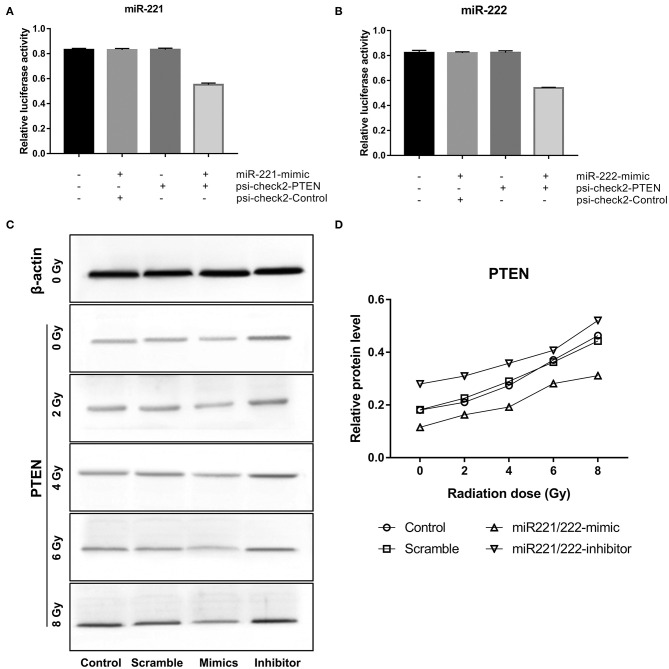
Phosphatase and tensin homolog (PTEN) is a target gene of *miR-221* and *miR-222* in IOMM-Lee cells. **(A,B)** Significantly decreased luciferase activities were revealed after the cotransfection of *miR-221* mimics or *miR-222* mimics and psi-check2-PTEN in dual luciferase reporter assay. **(C,D)** Western blot analysis exhibited that the expression of PTEN was positively correlated with radiation dose but negatively correlated with *miR-221/222* expression.

Dual luciferase reporter assay revealed that cotransfection of *miR-221* or *miR-222* mimics with psi-check2-PTEN significantly decreased luciferase activity compared to scramble or control-treated cells (*miR-221*: *P* < 0.0001; *miR-222*: *P* < 0.0001) (Figures 2A,B). Western blot analysis showed that PTEN was upregulated gradually as the *miR-221/222* expression level decreased or the radiation dose increased (Figures 2C,D). All these data demonstrated that PTEN is a target gene of the *miR-221/222* cluster.

### Downregulation of *miR-221/222* Expression and Irradiation Suppress Tumor Growth *in vivo*

**Figure 3:**
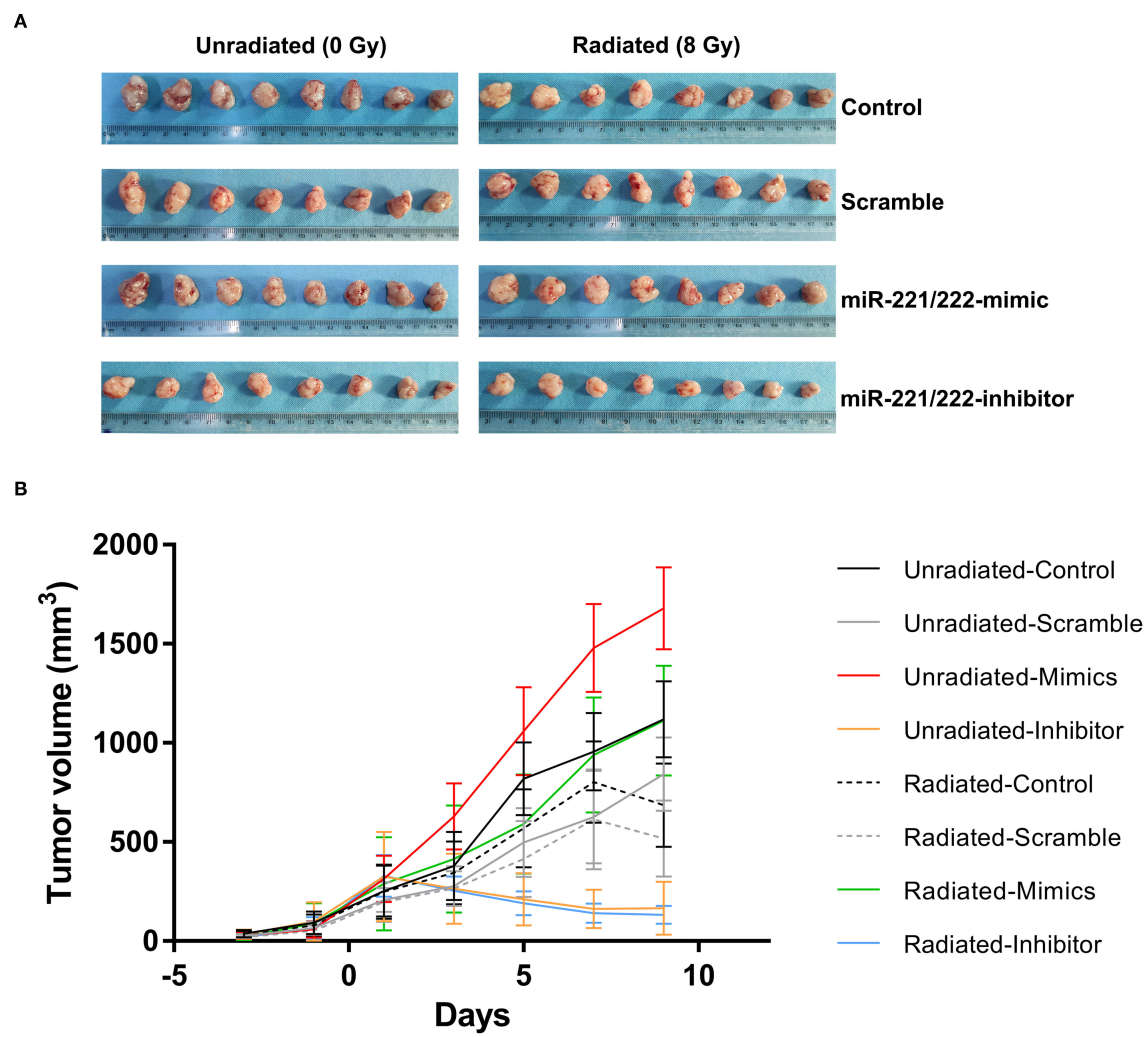
Downregulating *miR-221/222* expression along with radiation suppresses subcutaneous tumor growth *in vivo***. (A)** Exhibits the xenograft tumors from different treatment subgroups. **(B)** Reveals that both inhibition of the *miR-221/222* expression level and ionizing radiation significantly suppress tumor growth in nude mice.

Dramatic reductions in tumor volume were observed in irradiated control (38.66%), scramble (38.56%), *miR-221/222*-mimic (33.78%), and *miR-221/222*-inhibitor (20.08%) groups as compared with their respective unirradiated counterparts (Figure 3), indicating a significant inhibitory effect of irradiation on the volume of subcutaneous IOMM-Lee xenografts in nude mice (*P* < 0.0001, partial η^2^ = 0.474). For unirradiated animals, tumor volume in the *miR-221/222-*inhibitor group decreased by 85.21, 80.38, and 90.15% as compared with the control, scramble, and *miR-221/222-*mimic groups. The corresponding reduction rates in irradiation groups were 80.74, 74.48, and 88.11%. These suggest that the tumor volume can be suppressed by inhibiting the expression of *miR-221/222 in vivo* (*P* < 0.0001, partial η^2^ = 0.862). Furthermore, these two treatments have a synergistic effect on preventing tumor growth *in vivo* (*P* = 0.002, partial η^2^ = 0.232).

In addition to further proving these abovementioned results, multiple comparisons of tumor volumes at the last measure also revealed that no significant difference between the radiated-mimics group and the unradiated-control or unradiated-scramble group, indicating the antagonistic effect between radiation and upregulated *miR-221/222* expression *in vivo* (Table 8). A same situation was also found in the comparison between the unradiated-inhibitor group and the radiated-inhibitor group (Table 8). This may be explained by the observation that the effect of inhibiting the *miR-221/222* expression on preventing tumor progression has already been too obvious to reflect the effect of radiation.

**Table 8:** Time-varing tumor volumes of IOMM-Lee xenograft tumors in various treatment groups.

**Days**	**Description**	**Unradiated groups (Mean ± Standard deviation)**				**Radiated GROUPS (Mean ± Standard deviation)**			
		**Control**	**Scramble**	**Mimics**	**Inhibitor**	**Control**	**Scramble**	**Mimics**	**Inhibitor**
−3	1st Pre-treat	37.046 ± 18.881	25.701 ± 10.108	27.921 ± 13.966	34.697 ± 23.985	32.867 ± 18.433	18.493 ± 5.085	20.396 ± 15.011	20.432 ± 15.302
−1	2nd Pre-treat	92.979 ± 57.307	65.933 ± 33.077	59.935 ± 42.045	99.533 ± 97.297	78.893 ± 55.275	52.566 ± 25.984	89.624 ± 103.919	64.602 ± 57.082
0	1st -injection								
1	1st-measure	252.025 ± 129.423	207.469 ± 60.287	313.998 ± 116.227	325.252 ± 224.913	248.363 ± 137.236	198.245 ± 100.424	288.830 ± 235.379	329.696 ± 104.858
2	1st -IR								
3	2nd-measure	378.611 ± 171.727	277.653 ± 100.420	628.602 ± 167.446	264.124 ± 176.213	344.355 ± 158.887	266.415 ± 88.061	414.844 ± 269.869	253.916 ± 71.315
4	2nd -injection								
5	3rd-measure	819.921 ± 184.435	497.531 ± 173.248	1059.213 ± 222.273	211.468 ± 132.266	569.421 ± 196.957	414.461 ± 192.684	590.938 ± 250.908	190.157 ± 60.905
6	2nd -IR								
7	4th-measure	956.500 ± 195.499	626.219 ± 233.596	1478.570 ± 223.009	162.200 ± 97.474	801.723 ± 206.409	613.421 ± 252.433	939.645 ± 290.516	140.574 ± 47.782
8	3rd -injection								
9	5th-measure	1118.742 ± 191.996	843.166 ± 185.583	1679.081 ± 206.211	165.412 ± 134.343	686.242 ± 211.075	518.079 ± 192.757	1111.826 ± 277.750	132.189 ± 44.762
**Multiple comparisons (the 5th-measure)**, ***P*****-value**									
Unradiated-Control			0.157	<0.0001*	<0.0001*	0.001*	<0.0001*	1.000	<0.0001*
Unradiated-Scramble				<0.0001*	<0.0001*	1.000	0.035*	0.191	<0.0001*
Unradiated-Mimics					<0.0001*	<0.0001*	<0.0001*	<0.0001*	<0.0001*
Unradiated-Inhibitor						<0.0001*	0.014*	<0.0001*	1.000
Radiated-Control							1.000	0.001*	<0.0001*
Radiated-Scramble								<0.0001*	0.005*
Radiated-Mimics									<0.0001*

*IR, ionizing radiation; Pre-treat, Pre-treatment*.

* *Indicates statistical significance, and relevant P values are emphasized in bold*.

### Downregulation of *miR-221/222* Expression and Irradiation Promote the Expression of PTEN *in vivo*

**Figure 4:**
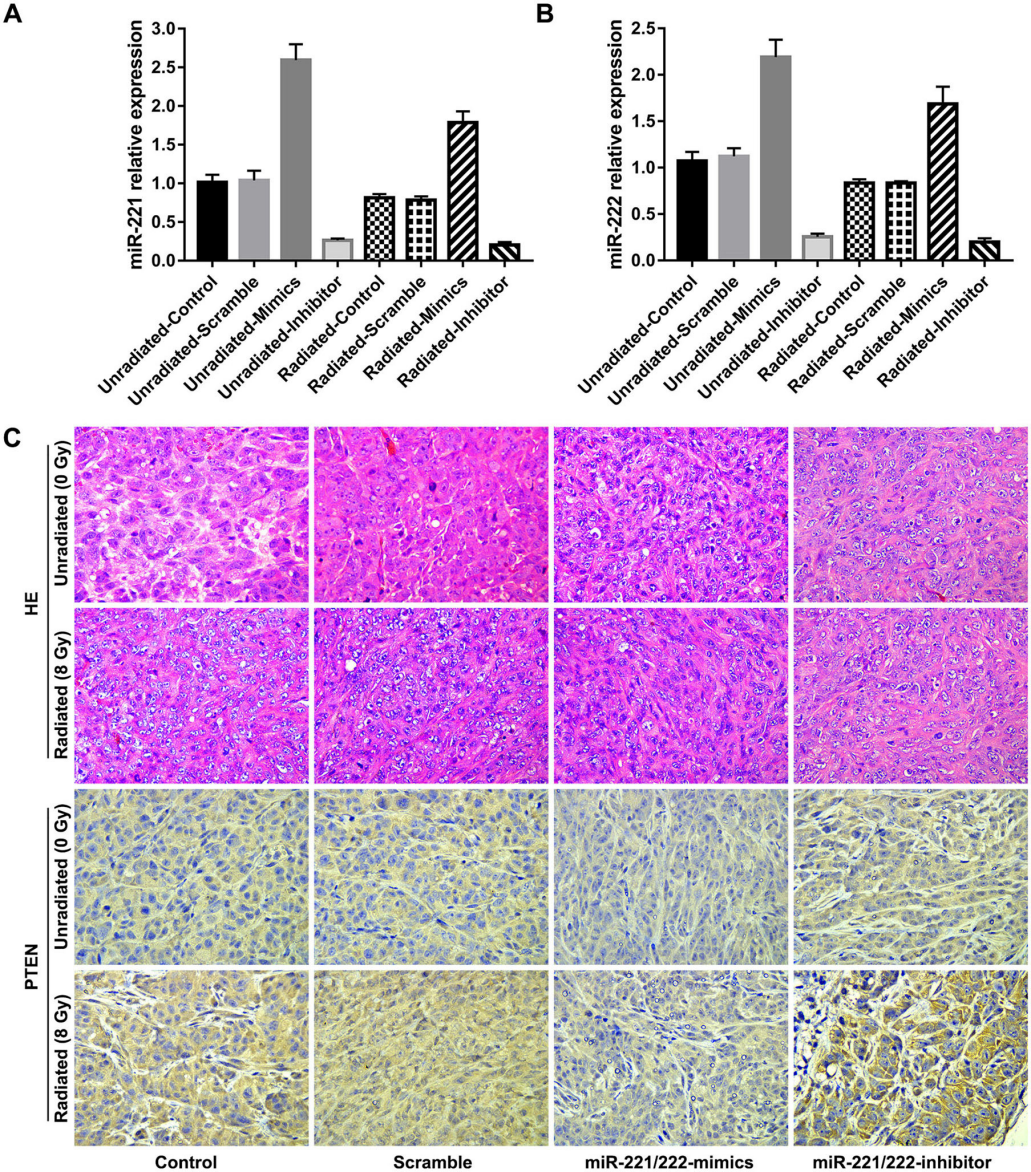
Downregulation of *miR-221/222* expression and ionizing radiation promote the expression of phosphatase and tensin homolog (PTEN) *in vivo*. **(A,B)** Show the relative expressions of *miR-221* and *miR-222* in subcutaneous tumors of different subgroups, respectively. **(C)** Exhibits immunohistochemical staining for PTEN (bottom, 400 ×) and their corresponding H&E staining (top, 400 ×) of tissue sections from each subgroup.

Immunohistochemical analysis of tissue sections of xenografts reflects a gradually increased immunoreactivity of PTEN as the *miR-221/222* expression decreases (Figure 4); meanwhile, tissue sections from radiation-treated xenografts exhibited higher expression levels of PTEN compared to their corresponding unirradiated counterparts (Figure 4). These are consistent with the results of *in vitro* studies.

## Discussion

In the present study, both radiation dose and expression level of *miR-221/222* significantly contributed to the regulation of the proliferation, colony formation, apoptosis, invasiveness, and subcutaneous xenografts of IOMM-Lee cells with significant interactions present, whereas they significantly regulated the sub-G0/G1 population without an interaction (Table 2). Increasing the radiation dose and downregulating *miR-221/222* expression level can synergistically inhibit the proliferation and colony formation, prevent subcutaneous xenografts progression, and promote the apoptosis of IOMM-Lee cells, while they antagonistically regulate the cell invasion (Tables 1, 2, 8 and Figures 1, 3). In addition, inhibiting the *miR-221/222* expression in IOMM-Lee cell can promote its radiosensitivity (Table 7). Consequently, downregulating the expression level of *miR-221/222* can promote the strengths of radiation and circumvent its weaknesses in IOMM-Lee cell treatment.

### Paradoxical Effects of Ionizing Radiation on IOMM-Lee Cells

The radiation dose-dependent apoptosis-promoting and proliferation-inhibiting effects of radiation on IOMM-Lee cells provide theoretical bases for utilizing postoperative radiation therapy to control the growth of residual or recurrent meningiomas in the clinic (Tables 1–3, 8, Figures 1C–E, 3). However, the radiation-induced invasiveness of IOMM-Lee cells may explain the unsatisfactory recurrence-free survival or even some toxicities of clinical adjuvant radiotherapy (Tables 1–3 and Figure 1H).

It has been revealed in several cancer cells (including breast, lung, and liver cancer, and glioma cells) that ionizing radiation (IR) enhances their migratory and invasive properties by inducing the epithelial–mesenchymal transition (EMT) (35–40). This IR-induced EMT is mediated by EMT-inducing transcription factors (EMT-TFs) (e.g., Snail, ZEB, and Twist families) that are activated by a network of signaling pathways (41–44). These EMT-TFs possess two potentials in cancer cells: (1) prometastatic potential—the aforementioned IR-enhanced migration and invasiveness reflect their prometastatic role. They regulate the expression level of proteins that is implicated in cell polarity, cytoskeletal structural maintenance, cell–cell contact, and extracellular matrix degradation, and they suppress key epithelial genes (e.g., E-cadherin) (41–44); (2) oncogenic potential: they are implicated in inducing malignant transformation (41, 45), stemness properties (41, 45), and oncogenic metabolism (41, 44). Hence, it is logical to assume that the present radiation-enhanced invasiveness of IOMM-Lee cells may be caused by the IR-induced EMT. In addition, we revealed in our previous clinical study that malignant progressed atypical meningiomas are more likely to exhibit low connexin 43 expression in their preradiotherapeutic tissues (46). Malignant transformation is one of the toxicities of radiotherapy in meningiomas (2). Connexin 43, the most abundant connexin isoform in the central nervous system (47, 48), oligomerizes to form gap junctions between adjacent meningioma cells (49, 50). These two points, as well as the present radiation-induced invasiveness of IOMM-Lee cells, all correspond to the prometastatic and oncogenic capacities of EMT-TFs. Al-Mefty et.al discovered the same complex genetic alterations that they saw in histologically higher-grade meningiomas already apparent in the early, benign stages of those tumors (51). Arishima et al. reported that different subtypes of meningiomas express different levels of connexin 43 (52). These findings raises the possibility that meningioma cells' inherent expression levels of certain moleculars and the intrinsic regulation level of EMT-TFs may determine whether this meningioma will undergo invasiveness enhancement, tumor recurrence, or malignant progression after radiotherapy.

### Downregulation of *miR-221/222* Expression Enhances the Apoptosis-Promoting Effect and Proliferation-Inhibiting Effect of Radiation and Promotes Radiosensitivity of IOMM-Lee Cells

The radiosensitization of downregulating the *miR-221/222* cluster has been certified in several human tumors: Zhang et al. successively discovered that tumor radiosensitivity could be promoted by the knockdown of *miR-221* and *miR-222* in gastric cancer cell line SGC7901 (31) and glioblastoma cell line U251, and demonstrated that PTEN is a target gene of the *miR-221/222* cluster (31); Sun and Khoshinani confirmed that *miR-221* (33) and *miR-222* (32) mediated the radiosensitivity of colorectal cancer cells by regulating PTEN, respectively; consistent results were reported by Wu and his colleague in their study of nasopharyngeal carcinoma (34). The radiosensitivity enhancement of *miR-221/222* downregulation and PTEN as the target gene of these two miRNAs were also confirmed in our present study of IOMM-Lee meningioma cells.

The PTEN gene, located at 10q23.3, was identified as one of the most commonly mutated tumor suppressor in human cancers, second only to p53 (53). Its encoded PTEN protein exhibits phosphoinositide 3-phosphatase activity toward phosphatidylinositol 3,4,5-trisphosphate and antagonizes phosphatidylinositol 3-kinase (PI3K) functions to negatively regulate cell proliferation and promote cell apoptosis (54). Loss-of-function mutations in the PTEN gene result in the inactivation of the PTEN protein, which further gives rise to oncogenic transformation of cells, resistance, and relapse in response to conventional therapeutic agents (55, 56).

IR exerts its therapeutic effect mainly by generating DNA damages (57). These IR-induced DNA damages, primarily double-strand breaks, trigger a number of DNA damage response and repair signaling cascades and subsequently phosphorylate p53 protein (58–61). Activated p53 upregulates the transcriptional and translational levels of several genes (including PTEN) to cause cell cycle arrest, apoptosis, autophagy, or senescence according to the severity of the DNA damage and the cell type (42, 62–65). Meanwhile, accumulation of PTEN, in turn, remarkably enhances p53 DNA binding and transcriptional activity by interacting with its C-terminal domain (66). Briefly, IR can induce PTEN accumulation to facilitate its therapeutic effects in some tumors. H460 cells obtained enhanced PTEN expression after irradiation in Il Lae Jung's previous research of nonsmall cell lung cancer (67). Similarly, the present radiation dose-dependent increase in PTEN in the IOMM-Lee cells suggests that the abovementioned mechanisms were activated during radiotherapy in meningiomas. Moreover, improved radiotherapeutic response in meningiomas can be achieved by further upregulation of PTEN through inhibiting the miR-221/222 expression.

The radiosensitization of PTEN has also been reported in previous literature: Rosser et al. identified forced expression of PTEN as a valuable approach to achieve radiosensitization in prostate cancer cells (68); multiple studies confirmed that the radioresistance of nasopharyngeal carcinoma could be enhanced by suppressing the expression of PTEN (69–71); consistent conclusions were obtained in the corresponding researches of non-small cell lung carcinoma (72), hepatocellular carcinoma (73), and esophageal cancer (74). Accordingly, the present observations indicate that the radiosensitization of *miR-221/222* inhibition in IOMM-Lee cells was achieved by its further upregulation of PTEN expression on the basis of IR-induced PTEN accumulation.

### Downregulation of *miR-221/222* Expression Can Reverse Radiation-Induced Cell Invasiveness

The IR-induced invasiveness of IOMM-Lee cells enhanced significantly as radiation dosage increased. Downregulation of miR-221/222 could promote the expression of PTEN and reverse the IR-enhanced cell invasiveness. As previously described, the IR-enhanced cell invasiveness is associated with EMT. Aside from their abovementioned radiosensitivity-regulatory effect, *miR-221* and *miR-222* have also been revealed to promote EMT (75) and increase migration and invasion in several other tumors (76). As a target of the miR-221/222 cluster, PTEN has been verified to possess the ability of reversing EMT in Jin's radioresistant esophageal cancer cells study (74). Therefore, it is conceivable that miR-221/222 downregulation reverses the radiation-induced cell invasiveness and is achieved by the EMT-reversion effect of accumulated PTEN. However, the underlying mechanisms of PTEN-regulated EMT in meningiomas require further investigations.

### Dose Rate of Irradiation Affects Cell Cycle Distribution of IOMM-Lee

In previous studies of IOMM-Lee cells, (1) Gogineni et al. indicated that radiation treatment (7 Gy) induced G2/M cell cycle arrest and a resultant decrease in the G0/G1 or S phase when evaluated against the unirradiated cells, as well as an insignificant cell-death-promoting effect (77). (2) However, by comparing the 5-Gy irradiated cells with unirradiated cells, Winson et al. exhibited a cell cycle distribution consist of an increased G0/G1, a decreased S, and an increased G2/M population, accompanied by an increment in apoptosis rate (78). The present results of radiation-induced G0/G1 cell cycle arrest and apoptosis-promoting effect are consistent with Winson's research while opposite to Gogineni's study (Tables 1–4 and Figures 1D–G). To determine the underlying causes of these opposites, we compared the data and discovered that the main difference is the dose rate, which was 0.71 and 3.2 Gy/min in the Gogineni's study (77) and the present study, respectively, while Winson et al. did not provide theirs (78).

According to Hall's revised and updated illustration of the dose-rate effect (79), the dose–response curve becomes progressively shallower as the dose rate reduces, indicating an increment in sublethal damage repair. Cells rest on their cell cycle phase without progression. However, a further reduction in dose rate in a limited range allows cells to progress through the cycle and accumulate in G2, resulting in the inverse dose-rate effect. The critical dose rate of IOMM-Lee initiating this effect has not been determined. With a higher dose rate in the present study, the capability of cells to repair sublethal damage was restrained, which further leads to an increase in apoptotic rate with a fixed cell cycle distribution. The dose rate used by Gogineni et al. is lower, which might have triggered the inverse dose-rate effect. This dose rate might not significantly increase cell death but have gradually accumulated cells to rest on G2 phase, and these may explain their cell cycle and apoptosis results.

In Kurpinski's research of differential effects of X-rays on human mesenchymal stem cells (80), it is proved that X-ray at a high dose rate (1 Gy/min) induces a significant increase in population of G0/G1 phase, a decrease in S phase, and no significant changes in G2/M phase in comparison with a low dose rate counterpart (0.1 Gy/min). We observed that the sub-G0/G1 population, which is referred to as an indicator of cell death, increased following a high dose rate radiation in IOMM-Lee cell (Tables 1, 2, 4 and Figures 1F,G). Combine with the dose-rate effect, these indicate that, within certain range, a higher dose-rate radiation treatment induces G0/G1 arrest and a relevant increased sub-G0/G1 population.

## Limitations

It is noteworthy that the present research is based only on one single meningioma cell line IOMM-Lee, which may not comprehensively reflect other cell lines. Acquisition of other meningioma cell lines is beyond our ability, and the corresponding assays should be performed for comprehensive evaluation.

## Conclusion

Radiation inhibits proliferation and promotes apoptosis and invasiveness in IOMM-Lee cells. Downregulating *miR-221/222* expression can reverse this radiation-induced cell invasiveness while enhancing the apoptosis-promoting and proliferation-inhibiting effects of radiation and promoting cell radiosensitivity. Meanwhile, the dose rate of irradiation was also revealed to affect cell cycle distribution and cell apoptosis of IOMM-Lee. A high dose-rate irradiation induces G0/G1 cell cycle arrest and apoptosis-promoting effect. These findings suggest that the downregulation of *miR-221/222* is a promising method of improving radiotherapeutic efficacy and preventing postradiotherapeutic tumor recurrence. Future investigations of meningioma cells may focus on the interaction mechanisms between *miR-221/222* and IR-induced EMT and EMT-TFs, which may improve the understanding of radiotherapeutic toxicities and achieve more effective toxicity avoidance.

## Data Availability Statement

The datasets generated for this study can be found in the article/supplementary material.

## Ethics Statement

The animal study was reviewed and approved by Animal Welfare Ethics Committee of Beijing Neurosurgical Institute (202001014).

## Author Contributions

QZ and L-RS: experimental implementation and acquisition of data. QZ, L-RS, X-LH, LW, G-BZ, S-YH, H-WJ, and C-LK: analysis and interpretation of data. QZ: drafting the article. J-TZ and G-JJ: approved the final version of the manuscript on behalf of all authors. J-TZ, G-JJ, WJ, ZW, LW, and B-NX: administrative, technical, and material support. All authors: conception and experimental design, critically revising the article, reviewed submitted version of manuscript, and study supervision.

## Conflict of Interest

The authors declare that the research was conducted in the absence of any commercial or financial relationships that could be construed as a potential conflict of interest.

## Abbreviations

DMEM: Dulbecco's modified Eagle's medium
FACS: fluorescence-activated cell-sorting
miR: microRNA
SER: sensitization enhancement ratio
SF: survival fraction.
